# Amorphous MoTe_x_ Nanomaterials Promote Visible-Light Co-Catalytic Degradation of Methylene Blue

**DOI:** 10.3390/ma18143388

**Published:** 2025-07-18

**Authors:** Zhen Zhang, Bin Liu, Jian Zhou, Zhimei Sun

**Affiliations:** 1 School of Materials Science and Engineering, Beihang University, Beijing 100191, China; by1901053@buaa.edu.cn (Z.Z.); jzhou@buaa.edu.cn (J.Z.); 2 National Key Laboratory of Spintronics, Hangzhou International Innovation Institute, Beihang University, Hangzhou 311115, China; binliu@buaa.edu.cn; 3 State Key Laboratory of Materials for Integrated Circuits, Shanghai Institute of Microsystem and Information Technology, Chinese Academy of Sciences, 865 Changning Road, Shanghai 200050, China

**Keywords:** molybdenum telluride, nanomaterials, co-catalytic, amorphous materials, transition metal chalcogenides

## Abstract

To investigate the application potential of amorphous transition metal chalcogenides in catalysis, this study successfully synthesized amorphous molybdenum telluride (MoTe_x_) materials and systematically explored their structural characteristics, compositional modulation, and catalytic performance. Experimental results indicate that the synthesized amorphous system consists of particles of approximately 200–300 nm in size. This distinct microstructure facilitates the exposure of abundant active sites and enhances physical adsorption capacity. The amorphous MoTe_2_/MoTe_3_ catalysts achieve an approximately 30%/40% degradation of methylene blue (MB) within 90 min, demonstrating significantly enhanced photocatalytic efficiency compared to that of crystalline MoTe_2_ (≈20% degradation under identical conditions). Furthermore, when integrated with titanium dioxide (TiO_2_), the composite exhibits exceptional co-catalytic performance, achieving a 90% degradation of MB within 90 min under visible-light irradiation, representing a catalytic efficiency improvement exceeding 160% compared to the results for pristine TiO_2_. Furthermore, through comparative analysis of the catalytic behavior and microstructural variations between amorphous MoTe_3_ (a-MoTe_3_) and MoTe_2_ (a-MoTe_2_), we observed that the catalytic activity of molybdenum tellurides exhibits a weak correlation with the tellurium content, with co-catalytic efficacy jointly governed by the density of the active sites and the physical adsorption properties. This research provides new methods and insights for the study and improvement of catalytic performance in chalcogenide materials.

## 1. Introduction

With the rapid development of modern industry, textile and paper manufacturing sectors generate substantial volumes of pollution containing methylene blue (MB) [1,2,3,4]. Due to its severe ecotoxicity and biodegradation resistance, the development of effective treatment strategies for MB-contaminated effluents is imperative to lessen long-term ecological impacts [5]. Therefore, the photocatalytic degradation of pollutants stands out as a highly promising strategy due to its environmental compatibility, high efficiency, and sustainability [6]. Among various photocatalysts, titanium dioxide (TiO_2_), a prototypical wide-bandgap semiconductor, has long dominated photocatalytic degradation research due to its exceptional stability, low toxicity, and cost-effectiveness [7,8,9,10]. However, owing to its broad bandgap structure, its practical catalytic efficiency remains constrained by limited visible-light utilization and rapid charge carrier recombination [11].

Recent advances have substantiated that heterojunction co-catalytic systems—incorporating metal oxides, ionic species, and multi-composite materials—constitute a viable approach for transcending the performance constraints inherent to single-component catalysts [11,12,13,14,15,16,17]. Thus, chalcogenide compounds have garnered widespread attention from researchers due to their unique electronic structure and exceptional catalytic performance [18]. Specifically, transition metal chalcogenides with a layered structure, such as MoS_2_ and MoSe_2_, are emerging as a new class of highly efficient co-catalysts [19,20]. Compared to their S- and Se-based materials, Te-based MoTe_2_ is a layered narrow-bandgap semiconductor (bandgap = 1.59 [21]) that has also attracted considerable interest in recent years due to its unique catalytic potential [22,23,24,25].

Building on research concerning MoS_2_ and similar materials, edges and basal planes containing vacancy defects are now generally considered highly active and can serve as centers for co-catalytic reactions [26,27,28,29,30,31]. However, the scarcity of catalytic sites has always been a key factor limiting the performance enhancement of transition metal chalcogenides [32,33,34,35]. Therefore, in recent years, research has been focused on the development and utilization of edge structures in MoS_2_ and other materials, aiming to further improve their catalytic efficiency. And amorphous materials demonstrate a promising direction [36,37,38].

Compared to long-range ordered crystalline materials, amorphous materials possess a unique disordered structure [39,40], which often leads to lattice distortions and unsaturated bonds within their structure [41,42]. The disordered atomic arrangement also enhances their reactivity and flexibility [43,44,45,46]. Amorphous materials can be regarded as the result of an interconnection of numerous edge states, which in turn provides a multitude of active sites and unique electronic properties [18,38,47]. In this context, amorphous chalcogenides emerge as a particularly promising candidate: their tunable bandgaps enable not only spectral response extension into the visible range but also directional interfacial charge migration that effectively mitigates recombination losses [6]. Moreover, compared to their crystalline chalcogenide counterparts, the amorphous phases can exhibit a higher density of catalytically active sites and distinct surface/interface architectures, which hold significant potential to synergistically enhance the photocatalytic efficiency of TiO_2_-based composite systems.

In this study, we developed an innovative synthesis strategy to obtain amorphous molybdenum telluride (MoTe_x_) materials through a room-temperature liquid-phase ion-mediated reaction. Subsequently, we composited the synthesized MoTe_x_ with TiO_2_ and systematically evaluated the catalytic/co-catalytic performance of the composite using methylene blue (MB) as a representative substrate. Additionally, the study investigated the factors contributing to the superior co-catalytic performance of amorphous molybdenum telluride materials through compositional tuning and pore structure analysis. The study not only achieved a significant enhancement of the visible-light photocatalytic degradation efficiency of TiO_2_ through a streamlined and efficient approach but also offers new insights for the design of future transition metal chalcogenide catalysts.

## 2. Materials and Methods

### 2.1. Materials

All the chemicals used were of analytical grade. Tellurium (Te) of 99.99% purity, ammonium molybdate ((NH_4_)_2_MoO_4_) of 99% purity, sodium-borohydride, NaBH_4_ of 99% purity, ethylenediaminetetraacetic acid (EDTA) of 99.5% purity, titanium dioxide (TiO_2_) of 99.99% purity, ethanol absolute of ≥99.5% purity was obtained from Shanghai Macklin (Shanghai, China), the crystalline MoTe_2_ reference material were purchased from Xinxi Technology (Shenzhen, China); All materials were used as received without further purification. Deionized water was employed throughout the study.

### 2.2. Preparation of the MoTe_x_ Materials

The synthetic strategy developed in this study presents distinct advantages compared to those of conventional protocols. Traditional amorphous-phase fabrication typically depends on high-temperature quenching processes, which not only demand energy-intensive conditions but also frequently involve toxic or hazardous precursor systems.

The product is obtained by combining tellurium anions with ammonium molybdate. The reaction mechanism is shown in the following formulas:

(1) $$2Te+4BH_{4}^{-}+7H_{2}O\to 2H{Te}^{-}+B_{4}O_{7}^{2-}+14H_{2}↑$$

(2) $$3H{Te}^{-}+(NH_{4}{)}_{2}MoO_{4}\overset{3H^{+}}{\to }Mo{Te}_{3}↓+2NH_{3}↑+4H_{2}O$$

Firstly, 20 mL of deionized water was added to a 100 mL three-neck flask, followed by the gradual addition of 2.0 g of NaBH_4_. While stirring, 30 mmol of elemental Te was slowly added, and the reaction mixture was stirred continuously for 1 h to ensure complete reaction. To the obtained telluride ion solution, 10 mmol of (NH_4_)_2_MoO_4_ was added, and the mixture was stirred at room temperature for an additional 1 h to ensure thorough reaction. After the reaction was complete, the mixture was transferred to a centrifuge tube and centrifuged at 5000 rpm for 10 min to separate the solid product. The washed solid product was then carefully transferred to a vacuum drying oven and dried at 60 °C for 12 h until completely dry. For specific details of the process, please refer to the Supplementary Materials.

### 2.3. Fabrication of MoTe_x_@TiO_2_ Nanocomposites

We took 50 mg of the prepared MoTe_x_ products and dispersed it in 20 mL of ultrapure water via ultrasound. We added the obtained suspension into 0.45 g of anatase TiO_2_ dispersion and stirred the mixture evenly. Then, we slowly poured the mixed suspension into the vacuum suction bottle for suction filtration. After suction filtration, we transferred the obtained film to a vacuum-drying oven and dried it at room temperature for 12 h to obtain MoTe_x_@TiO_2_ nanocomposites.

### 2.4. Characterizations of Materials

The morphological features and elemental compositions of the MoTe_x_ and MoTe_x_@TiO_2_ nanocomposites were analyzed using field emission scanning electron microscopy (FESEM, JEOL JSM7001F, Tokyo, Japan) equipped with energy-dispersive spectroscopy (EDS), and transmission electron microscopy (TEM, JEM-2010, Tokyo, Japan) was used to determine the amorphous state of the synthesized MoTe_x_. The zeta potential and particle size distribution of the MoTe_3_ dispersions were characterized via a nanoparticle size and zeta potential analyzer (Zetasizer Nano ZS, Malvern Panalytical, Malvern, UK). The phase transformation behavior of MoTe_3_ was characterized using differential scanning calorimetry (DSC). The phase identification and lattice parameters were determined through X-ray diffraction (XRD, Rigaku-D MAX-2550PC diffractometer using Cu Kα radiation with scanning steps of 0.02°, Tokyo, Japan). The bonding properties were analyzed using X-ray photoelectron spectroscopy (XPS, Thermo SCIENTIFIC ESCALAB Xi+, Waltham, MA, USA) equipped with a monochromatic Al Kα (1486.6 eV). The XPS data analysis was performed using Avantage software (version 5.967, Thermo Fisher Scientific, Waltham, MA, USA). Raman spectroscopy was employed for testing, utilizing an NRS-7100 Laser Raman Spectrometer (JASCO, Tokyo, Japan). The specific surface area and pore distribution of the samples were assessed using the Brunauer–Emmett–Teller (BET) method.

The photocatalytic performance of MoTe_x_@TiO_2_ was evaluated using MB. A 500 W Xe lamp (GXZ500, Shanghai Jiguang Special Lighting Electric Appliance Factory, Shanghai, China) was used for simulating sunlight. The distance from the lamp to the MB suspension liquid level was 15 cm. As is typical, 10.0 mg of MoTe_x_@TiO_2_ were added into an aqueous MB solution (50 mL, 10 mg/L), and then the MB photocatalytic degradation experiment was carried out under the irradiation of the Xe lamp. All photocatalytic experiments in this study incorporated a 60 min dark adsorption process to eliminate potential errors. At a certain time interval, a 3 mL suspension was withdrawn from the reaction, and the MB concentration was determined by a UV–VIS spectrophotometer (UV-1240 Shimadzu, Kyoto, Japan) at the wavelength of 664 nm. To reveal the photocatalytic mechanism, typical radical scavenger ethylene diamine tetra acetic acid (EDTA) was used in the reactions to verify the active species.

## 3. Results and Discussion

### 3.1. Morphology and Structure Characterization

**Figure 1:**
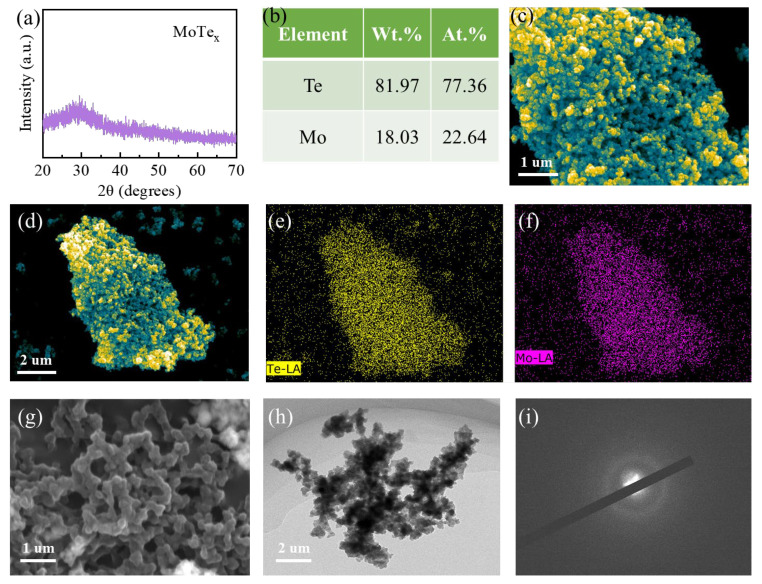
The characterization of MoTe_x_ using X-ray diffraction (XRD) and scanning electron microscopy (SEM). (**a**) XRD pattern of MoTe_3_. (**b**) The elemental distribution of MoTe_x_ based on energy-dispersive X-ray spectroscopy (EDS). (**c**) SEM image of MoTe_3_ particles. (**d**–**f**) The distribution of Te and Mo elements in MoTe_3_ particles. (**g**) Ultrasonically dispersed polymer-like MoTe_3_ particles. (**h**) Transmission electron microscopy (TEM) images of MoTe_3_. (**i**) Transmission electron microscopy electron diffraction pattern of MoTe_3_.

To confirm the phase and composition of the reaction products, we conducted a characterization and analysis. The obtained powder XRD pattern exhibited distinct amorphous characteristics, with a pronounced peak around 30 degrees, which is highly similar to that of other amorphous chalcogenides (Figure 1a). The absence of any sharp peaks in the XRD suggests that the product is a homogeneous amorphous material. Additionally, the suspended MoTe_x_ product manifested as particles, with some aggregation (Figure 1c). Energy-dispersive X-ray spectroscopy (EDS) was also employed to assess the elemental distribution within the materials (Figure 1b). It was observed that the distribution of Te and Mo elements in the sample was very uniform (Figure 1d–f), with a ratio close to 3:1. This stoichiometry is different from that of typical molybdenum telluride (crystalline MoTe_2_) and is more reflective of the characteristics of amorphous materials. Consequently, we designate this phase as a-MoTe_3_ in subsequent discussions. This preliminary characterization indicates that the synthesized amorphous MoTe_3_ exhibits characteristics typical of amorphous materials. Notably, this stoichiometry is related to the ionic valences of the two elements (Te (−2) and Mo (+6)), reflecting the credibility of the reaction mechanism in Formula (2).

After ultrasonic dispersion, the MoTe_3_ samples more intuitively exhibit an interconnected morphology (Figure 1g,h), in contrast to the typical monodispersed state of the amorphous particles [39]. The electron diffraction patterns further confirm the amorphous characteristics of the material. (Figure 1i) The microstructure of MoTe_x_ resembles a network composed of interconnected clusters, which demonstrates the uniqueness of the Mo–Te bonding in the material. It is likely to possess a bridging structure similar to that of α-MoS_x_. To further characterize the properties of the obtained MoTe_3_ particles, we performed characterization using a Zetasizer Nano ZS nanoparticle size analyzer (Malvern, UK).

**Figure 2:**
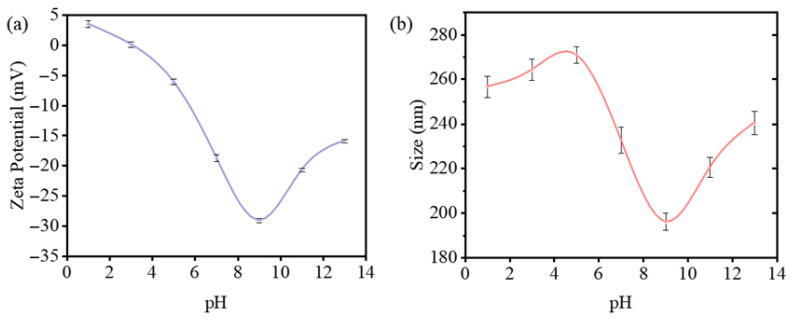
Characterization of MoTe_3_ particles: (**a**) zeta potential; (**b**) particle size distribution.

The results (Figure 2) show that the material’s zeta potential changes significantly with changes in pH. At pH 9.0, the zeta potential reaches approximately −30 mV, indicating the formation of a relatively stable dispersion. As pH decreases, the absolute zeta potential value rapidly approaches zero, accompanied by a clear decrease in system stability—consistent with our experimental observations. At pH 14.0, while stability decreases (zeta potential ≈ −15 mV), rapid aggregation would not occur. The characterization also revealed particle size distribution in the liquid phase. The particle size fluctuates with pH changes, reaching a minimum of about 190 nm at pH 9.0. The maximum diameter under different pH conditions remains below 300 nm, which is generally consistent with our SEM and TEM observations, confirming that the material exhibits typical nanoparticle properties.

Based on previous studies, it is generally believed that amorphous chalcogenide compounds often possess a polymer-like cross-linked network structure [37]. It is reasonable to speculate that the MoTe_3_ we synthesized may also possess similar properties. To address this, we characterized the chemical states of the Mo and Te elements in the samples using X-ray photoelectron spectroscopy (XPS) and Raman spectroscopy.

**Figure 3:**
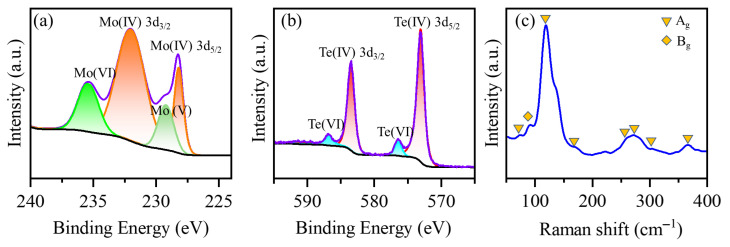
(**a**) XPS spectrum of Mo 3d. Mo(VI): bright green curve; Mo(IV): orange curve; Mo(V): green curve. (**b**) XPS spectrum of Te 3d. Te(VI): bright blue curve; Te(IV): red curve. (**c**) Raman spectrum of MoTe_3_.

The peak analysis for the Mo element revealed that the Mo 3d_5/2_ peak occurs at 228.2 eV, and the Mo 3d_3/2_ peak is observed at 232.1 eV, characteristic of Mo(IV) (Figure 3a, orange curve). The presence of Mo(IV) indicates that amorphous MoTe_3_ contains Mo elements with chemical states similar to those in crystalline MoTe_2_. However, a distinct Mo(VI) peak is observed at 235.4 eV (Figure 3a, green curve). Although the Mo in our starting material (ammonium molybdate) was already in the +6 oxidation state, the appearance of this peak typically suggests the oxidation of Mo in the material [48]. This phenomenon is commonly observed in two-dimensional MoTe_2_. In our obtained nanoparticles, due to their amorphous nature, Mo elements are more exposed at the surface and thus more susceptible to oxidation, which may affect subsequent catalytic performance. Additionally, we note the presence of a Mo(V) peak at 229.2 eV, which, according to literature reports, indicates the existence of Mo–O bonds [49], further confirming the partial oxidation of the material. However, compared to typical MoTe_2_, the 3d_5/2_ peak of Mo shifts towards higher binding energy, indicating an increased trend for Mo to donate electrons. Considering that the intrinsic electronegativity of the elements does not differ, this suggests an increased Mo–Te bond formation. For the Te element, the XPS spectrum exhibits two distinct doublets. The first doublet at 573 eV and 583.9 eV corresponds to Te(IV), representing characteristic Mo–Te bonding [49], confirming that the product is predominantly composed of Mo–Te bonds. Additionally, a weaker doublet is observed at 577.0 eV and 587 eV, which, after analysis, is attributed to Te(VI) (Te–O). As previously discussed regarding the partial oxidation of Mo, the Te element also shows signs of oxidation. This is particularly evident considering that XPS is a surface-sensitive technique that is more responsive to surface oxidation in nanomaterials due to their high specific surface area. Combining the XPS results of both Mo and Te, we conclude that the obtained MoTe_3_ material primarily consists of Mo–Te bonding. However, the high surface area of the nanomaterial leads to some degree of surface oxidation, which is a typical characteristic of small-sized materials. Based on XPS analysis, we also calculated the atomic ratio of Te to Mo elements, which is in good agreement with the results from EDS (Table S1).

Raman spectroscopy with a 532 nm excitation laser was employed to further evaluate the chemical states of MoTe_3_ (Figure 3c). The Raman spectrum exhibited characteristic peaks at 84.3, 102.7, 130.3, 161.3, 252.2, and 264.8 cm^−1^, which are relatively close to the reported Raman modes of monoclinic MoTe_2_. However, it is noteworthy that the peaks at typical Raman positions (e.g., 161.3 cm^−1^) all appear broad rather than sharp, indicating the low crystallinity that is characteristic of amorphous materials [48,50]. These Raman results demonstrate that the obtained MoTe_3_ maintains a bonding configuration dominated by Mo–Te interactions, yet distinctly differs from typical crystalline MoTe_2_. The data analysis above indicates that the amorphous MoTe_3_ we prepared displays distinct Mo–Te bonding, but compared to regular MoTe_2_, the number of bonds has increased. Considering its morphological characteristics, it is sufficient to conclude that it also possesses the chain-like structure typical of amorphous chalcogenide compounds [51]. Such long-range disordered structural configurations, coupled with high specific surface areas, undoubtedly confer inherent advantages to their catalytic performance.

Leveraging the compositional flexibility inherent to amorphous materials, we attempted to synthesize amorphous MoTe_2_ from the MoTe_3_ precursor, thereby enabling direct performance comparisons with that of crystalline MoTe_2_. This comparative approach facilitates systematic investigation into the role of chalcogenides within co-catalytic systems, particularly their influence on active site density. Firstly, we conducted differential scanning calorimetry (DSC) characterization on the amorphous MoTe_3_ (Figure S1). A distinct endothermic peak was observed around 450 °C, indicating a significant change in the properties of the amorphous MoTe_3_ at this temperature. Above 500 °C, the material exhibited significant weight loss, which, in conjunction with the characteristics of Te, suggests that the volatilization of Te elements began to occur. To further validate our hypothesis regarding the transition process, we selected several typical time points to analyze the morphology and XRD patterns of the samples.

**Figure 4:**
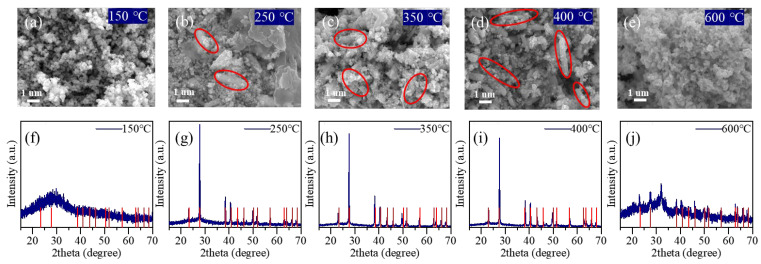
SEM images of MoTe_x_ materials at different temperatures: (**a**) 150 °C; (**b**) 250 °C; (**c**) 350 °C; (**d**) 400 °C; (**e**) 600 °C. XRD pattern of MoTe_x_ materials at different temperatures: (**f**) 150 °C; (**g**) 250 °C; (**h**) 350 °C; (**i**) 400 °C; (**j**) 600 °C. The red vertical lines in the XRD pattern correspond to the reference diffraction peaks of elemental tellurium (ICSD No. 98-002-3067), which serve as standard markers for phase identification.

The XRD patterns at 150 °C still maintained the typical characteristics of amorphous materials, and the microstructure of the samples showed almost no change (Figure 4a,f). This indicates that the amorphous material we prepared exhibits excellent thermal stability. At 250 °C, Te nanocrystals began to precipitate from the sample (red circles). The XRD patterns also showed distinct sharp peaks corresponding to those of elemental Te (Figure 4b,g). At 350 °C and 400 °C, the precipitation of Te became more pronounced, with an increasing quantity and growing size of the nanocrystals. The XRD patterns still contained a significant number of patterns from elemental Te (Figure 4c,d,h,i). When the temperature reached 600 °C, the microstructure of the sample returned to a spherical cluster form, and the elemental Te nanocrystals had almost completely volatilized (Figure 4e). The XRD tests also confirmed this, with the sharp peaks almost disappearing and the overall pattern again exhibiting significant amorphous characteristics (Figure 4j). EDS analysis revealed a Mo:Te atomic ratio of 1:2, confirming the formation of amorphous MoTe_2_ (Table S2). The transformation characteristics are consistent with our expectations, and it is interesting to note that the MoTe_2_ product remains amorphous. This also confirms our inference regarding the structure of MoTe_3_. Given the discussion on the structure of amorphous MoTe_3_, it is indeed irrational for the cross-linked structure, which loses some Te atoms after the phase change, to directly transform into the typical crystalline MoTe_2_.

### 3.2. Photocatalyst Degradation of Methylene Blue

Researchers generally believe that in transition metal chalcogenides, particularly in their two-dimensional forms, the edge states possess a significant catalytic advantage, which is attributed to the exposed metal atoms [30]. Meanwhile, molybdenum chalcogenides demonstrate strong adsorption and enrichment capabilities toward specific organic dyes, rendering them particularly promising for photocatalytic degradation applications [52]. Therefore, the amorphous MoTe_3_ and MoTe_2_ materials synthesized in this study exhibit dual structural advantages: their unique disordered structure not only generates abundant microscopic “edge states” but also contributes to increase specific surface area and adsorption capacity. These synergistic characteristics theoretically predict superior catalytic performance. To verify the speculation regarding the catalytic properties of amorphous MoTe_x_, we selected typical methylene blue (MB) [53] as the photocatalytic degradation substrate. Simultaneously, based on previous literature studies suggesting the superior co-catalytic performance of molybdenum chalcogenides [52,54,55], we prepared MoTe_x_@TiO_2_ composite materials for catalytic testing (Figures S2 and S3) and benchmarked these against analogous systems to assess the enhancement of TiO_2_ photocatalytic performance induced by amorphous chalcogenides.

**Figure 5:**
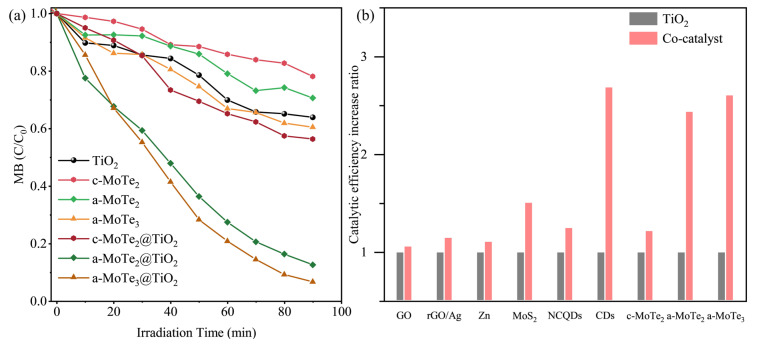
(**a**) The photocatalytic degradation efficiency of different catalytic materials for methylene blue (MB). (**b**) Comparative enhancement capabilities of different co-catalytic systems for the photocatalytic degradation efficiency of TiO_2_ [57,58,59,60,62,63]. More supplementary experimental conditions are shown in Table S4.

To investigate whether co-catalytic systems can extend the spectral response range of TiO_2_, catalytic experiments were conducted under visible light irradiation. Initial observations revealed that amorphous MoTe_3_ (a-MoTe_3_) and amorphous MoTe_2_ (a-MoTe_2_) exhibited activity in methylene blue (MB) degradation, achieving approximately 40% MB decomposition within 90 min, significantly surpassing the performance of crystalline MoTe_2_ (c-MoTe_2_). (Figure 5a) This efficiency level was nearly comparable to that of intrinsic anatase TiO_2_. Notably, however, the catalytic efficiency of TiO_2_ under visible light was substantially reduced compared to that under ultraviolet light, a limitation attributed to its inherent wide bandgap nature [56]. However, when MoTe_x_ is combined with TiO_2_, the catalytic efficiency of the composite significantly improves. While c-MoTe_2_@TiO_2_ exhibited comparatively inferior catalytic performance, it nonetheless achieved 40% methylene blue (MB) degradation within 90 min. Meanwhile, both a-MoTe_2_@TiO_2_ and a-MoTe_3_@TiO_2_ successfully decomposed about 90% of the MB substrate within 90 min, which demonstrates at least a 140% enhancement in catalytic degradation efficiency compared to that of isolated TiO_2_. To systematically evaluate the enhancement capabilities of various co-catalytic materials on TiO_2_, we conducted a comprehensive comparative analysis of analogous systems. Given the significant variations in the catalytic performance of TiO_2_ across different experimental systems, direct comparisons of degradation efficiency fail to isolate the intrinsic contributions of co-catalysts. To address this limitation, we normalized the literature-derived data by defining the intrinsic catalytic degradation efficiency of pristine TiO_2_ as 1.0 under every different experimental condition to achieve unity. Subsequently, the catalytic enhancement factor of each co-catalyst was calculated as the relative efficiency ratio compared to this normalized baseline (Figure 5b). The results demonstrate that conventional co-catalysts—including metallic species [57], two-dimensional materials [58], and crystalline MoS_2_ [59]—yielded less than a 50% improvement in the catalytic efficiency of TiO_2_. Notably, carbon nanodots (CDs) achieved a performance enhancement exceeding 160%, comparable to that of amorphous MoTe_x_ (a-MoTe_x_) [60]. However, compared to the intricate synthesis and composite integration processes required for CDs, a-MoTe_x_ emerges as a far more practical co-catalytic material, offering superior cost-to-performance efficiency in scalable manufacturing. Foreseeably, with optimized application conditions, this catalyst series will exhibit more significant potential. The improvement in performance, based on research into transition metal chalcogenides, should be attributed to the unique bonding structure of MoTe_x_, which can better absorb photons and separate the photo-produced carriers [61]. Supplementary experiments substantiated the viewpoint (Table S3). The addition of EDTA (a typical OH· scavenger) most significantly reduced the MB degradation efficiency across all three composite catalysts when compared to the effects of CHOONa and p-benzoquinone, indicating that the degradation of MB occurred through hole-mediated pathways via hydroxyl radical (OH) generation [60]. Thus, we acknowledge that the enhanced catalytic performance of TiO_2_ facilitated by MoTe_x_ arises primarily from the increased yield of hydroxyl radical (OH) species. Since the radical-mediated oxidative processes are fundamentally critical in air purification and antimicrobial disinfection, it is reasonable to believe that the MoTe_x_@TiO_2_ co-catalytic system could also demonstrate considerable potential in the aforementioned application domains.

**Figure 6:**
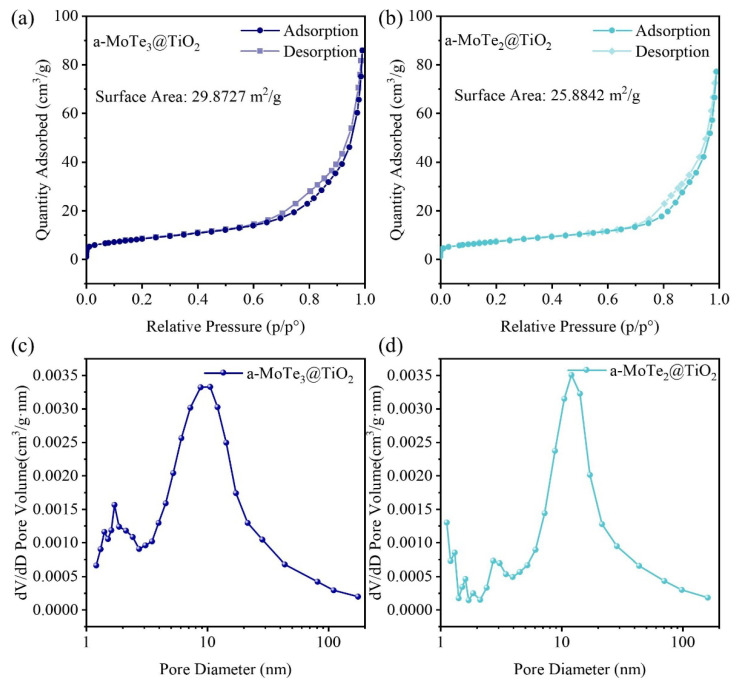
The nitrogen adsorption–desorption isotherms and pore size distributions of a-MoTe_3_@TiO_2_ and a-MoTe_2_@TiO_2_. Nitrogen adsorption–desorption isotherms of (**a**) a-MoTe_3_@TiO_2_ and (**b**) a-MoTe_2_@TiO_2_. Pore size distribution diagrams of (**c**) a-MoTe_3_@TiO_2_ and (**d**) a-MoTe_2_@TiO_2_.

The comparative analysis reveals that amorphous MoTe_x_ demonstrates substantial advantages over its crystalline MoTe_2_ counterparts, not only in regards to its intrinsic catalytic activity but particularly concerning its co-catalytic performance. Given their analogous degradation mechanisms (predominantly hydroxyl radical-mediated pathways), this enhanced efficacy fundamentally originates from its unique disordered structure, which provides increased active site density at equivalent loadings, thereby significantly amplifying overall catalytic capacity. It is also noticeable that the catalytic performance of a-MoTe_2_@TiO_2_ is slightly lower compared to that of a-MoTe_3_@TiO_2_. This suggests that the difference in Te content has an impact on the co-catalytic performance of MoTe. Given that hole scavengers exhibit comparable attenuation effects across different MoTe_x_ systems, the performance discrepancies likely originate from structural discrepancies at the microscopic level. These disparities fundamentally stem from differential adsorption capacities inherent to distinct amorphous compositions. To validate this hypothesis, we conducted systematic analyses of the composite catalysts’ specific surface areas and pore size distributions (Figure 6).

### 3.3. Specific Surface and Pore Size Analysis of MoTe_x_@TiO_2_

The nitrogen adsorption–desorption isotherms for both composite materials are relatively consistent, lacking a saturated adsorption platform, indicating that they are typical macroporous adsorbents. However, when comparing the specific surface areas of the two, it is observed that the surface area of a-MoTe_2_@TiO_2_ has decreased compared to that of a-MoTe_3_@TiO_2_, which aligns with our hypothesis (Figure 6a,b). Due to the reduction in Te atoms required for the amorphous network, the disordered structure of the a-MoTe_2_@TiO_2_ composite samples is inevitably simplified compared to that of a-MoTe_3_@TiO_2_, leading to a tendency to aggregate and stack, which in turn reduces the corresponding specific surface area. The distribution of pore quantity also supports a similar conclusion: compared to a-MoTe_3_@TiO_2_, a-MoTe_2_@TiO_2_ shows a significant reduction in the number of micropores (<2 nm), while there is almost no difference in the content of mesopores (Figure 6c,d), which is consistent with the changes in the material’s microstructure and composition.

It is well established that the specific surface area of a catalyst can significantly influences the rate of a reaction [64]. Given that the specific surface area difference between the two composite materials is about 15%, and their catalytic efficiencies after 90 min differ by less than 10%, we can infer that the performance differences between the two catalysts are likely more attributable to their microstructural differences rather than to changes in the chemical state or electronic structure due to the differences in the Te elements. This finding substantiates that for MoTe_x_ materials, both active site increase and specific surface area enhancement (directly governing adsorption capacity) constitute dual determinants of catalytic efficacy. Crucially, the distinctive disordered amorphous structure simultaneously amplifies both parameters through structural disordering. Thus, amorphous-based chalcogenides emerge as a promising frontier for next-generation catalytic material development.

## 4. Conclusions

In this study, amorphous MoTe_x_ materials with polymeric networks were synthesized via a room-temperature ionic approach. Hybridization with TiO_2_ revealed exceptional co-catalytic performance in the MoTe_x_ composites. Systematic comparison between a series of crystalline and amorphous MoTe_x_ materials further elucidated the mechanistic basis for their catalytic enhancement. The amorphous structure was demonstrated to concurrently increase both active site density and substrate adsorption capacity through its cross-linked configuration, thereby synergistically boosting photocatalytic degradation efficiency. These findings establish the use of amorphous chalcogenides as a developmental pathway for further optimizing transition metal dichalcogenide catalysts.

## Data Availability

The original contributions presented in this study are included in the article/Supplementary Materials. Further inquiries can be directed to the corresponding author.

## Supplementary Materials

The following supporting information can be downloaded at: https://www.mdpi.com/article/10.3390/ma18143388/s1, Experimental details; Table S1: The elemental distribution of MoTe_3_ based on XPS; Figure S1: DSC characterization of amorphous MoTe_3_ (the blue areas and numbers indicate the detection points selected); Table S2: The Mo–Te stoichiometric ratio in MoTex materials under varying temperatures; Figure S2: Scanning electron microscopy (SEM) characterization and energy dispersive spectrometer (EDS) mapping of the synthesized a-MoTe_3_/TiO_2_; Figure S3: Scanning electron microscopy (SEM) characterization and energy dispersive spectrometer (EDS) mapping of the synthesized a-MoTe_2_/TiO_2_. Table S3: Degradation efficiency comparison between composites catalyst series incorporating EDTA, CHOONa, and PBQ sacrificial agent and blank controls at 90 min. Table S4: Supplementary experimental conditions for different co-catalysts.
