# Supplementary material for: Amorphous MoTex Nanomaterials Promote Visible-Light Co-Catalytic Degradation of Methylene Blue
Source: Materials (Basel). 2025 Jul 18;18(14):3388. doi: 10.3390/ma18143388 (PMC12300420; doi:10.3390/ma18143388)
Supplement: Supplementary file 1 [file materials-18-03388-s001.zip › materials-3740260-supplementary.pdf]

Amorphous MoTex from Reversible Colloid Promote Visible-Light Co-Catalytic Degradation of Methylene Blue

Zhen Zhang [1], Bin Liu [2,3] Jian Zhou [1]and Zhimei Sun [1, *]

[1] School of Materials Science and Engineering, Beihang University, Beijing 100191, China.; by1901053@buaa.edu.cn; jzhou@buaa.edu.cn.
[2] National Key Laboratory of Spintronics, Hangzhou International Innovation Institute, Beihang University, Hangzhou 311115, China. binliu@buaa.edu.cn;
[3] State Key Laboratory of Materials for Integrated Circuits，Shanghai Institute of Microsystem and Information Technology，Chinese Academy of Sciences，865 Changning Road, Shanghai 200050 China. binliu@buaa.edu.cn;
* Correspondence: zmsun@buaa.edu.cn; Tel: 010-82317747 (o)

Experiment Details

Firstly, 20 mL of deionized water was added to a 100 mL three-neck flask, followed by the gradual addition of 2.0 g of $NaBH_4$. The solution was stirred using a magnetic stirrer until the $NaBH_4$ was completely dissolved. While stirring, 30 mmol of elemental Te was slowly added, and the reaction mixture was stirred continuously for 1 hour to ensure complete reaction. Upon completion of the reaction, the solution turned pale purple, indicating that the elemental sulfur had been successfully reduced to telluride ions (Te^{2-}).

To the obtained telluride ion solution, 10 mmol of $(NH_4)_2MoO_4$ was added, and the mixture was stirred at room temperature for an additional 1 hours to ensure thorough reaction. After the reaction was complete, the mixture was transferred to a centrifuge tube and centrifuged at 5000 rpm for 10 minutes to separate the solid product. The solid product obtained from centrifugation was washed three times with deionized water and anhydrous ethanol, each time using 50 mL, to remove any residual reactants and by-products. The washed solid product was then carefully transferred to a vacuum drying oven and dried at 60°C for 12 hours until completely dry. Through these steps, a pure target compound was obtained, which can subsequently be used for further characterization and analysis.

Table S1 The elemental distribution of $MoTe_3$ based on XPS

Element	At. %
**Te**	76.03
**Mo**	23.97

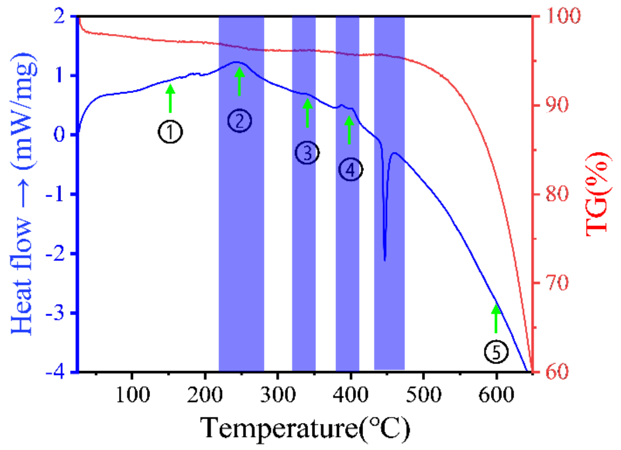

Figure S1. DSC characterization of amorphous MoTe3 (the blue areas and numbers indicate the detection points we selected).

Table S2. The Mo, Te stoichiometric ratio in MoTex materials under varying temperatures.

Temperature (°C)	150	250	350	400	600
**Te (at. %)**	77.3	75.4	78.5	78.9	67.1
**Mo (at. %)**	22.7	24.6	21.5	21.1	32.9

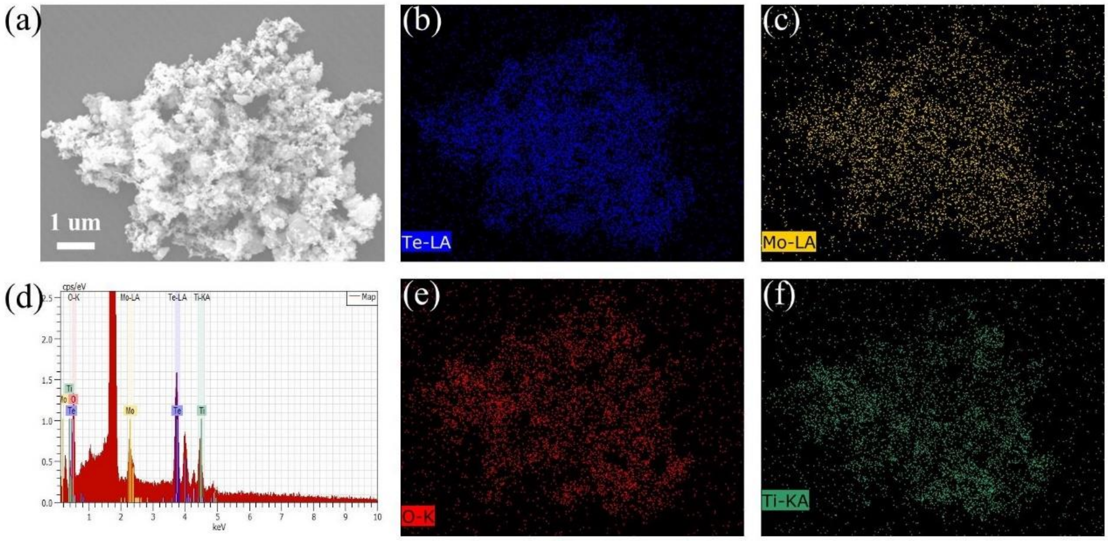

Figure S2. Scanning electron microscopy (SEM) characterization and energy dispersive spectrometer (EDS) mapping of the synthesized a-$MoTe_3/TiO_2$.

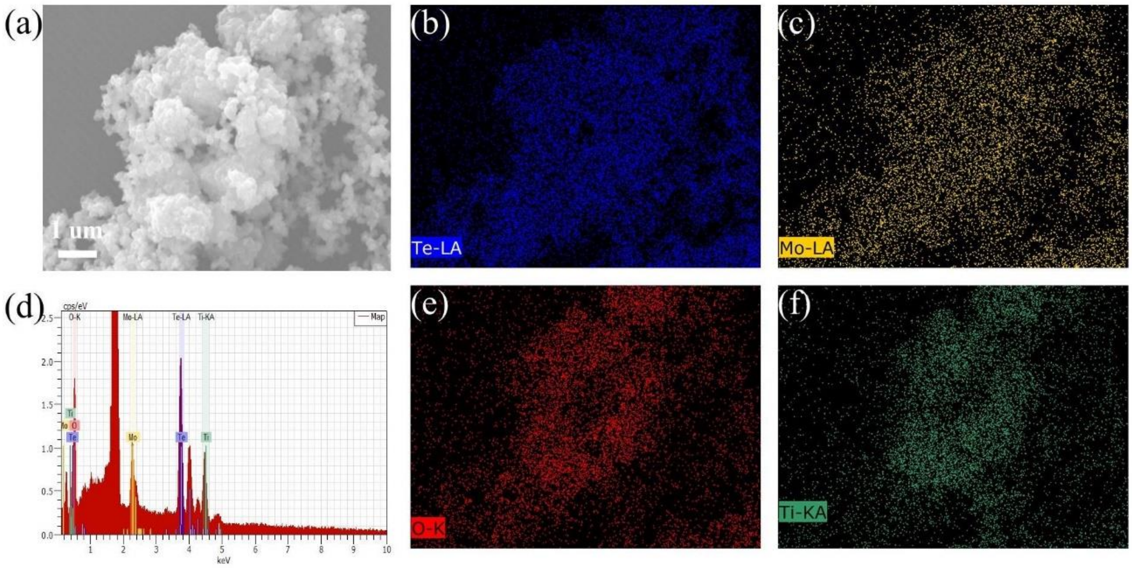

Figure S3. Scanning electron microscopy (SEM) characterization and energy dispersive spectrometer (EDS) mapping of the synthesized a-$MoTe_2/TiO_2$.

Table S3. Degradation efficiency comparison between composites catalyst series incorporating EDTA, CHOONa and PBQ sacrificial agent and blank controls at 90 min.

	$c-MoTe_2@TiO_2$		$a-MoTe_2@TiO_2$		$a-MoTe_3@TiO_2$	
90 min Degradation (%)	43.8	19.7 (EDTA)	87.5	38.7 (EDTA)	93.5	42.0 (EDTA)
		42.3 (CHOONa)		85.2 (CHOONa)		93.2 (CHOONa)
		35.5 (PBQ)		66.8 (PBQ)		74.9 (PBQ)

Table S4. Supplementary experimental conditions for different cocatalysts

catalyst	catalyst concentration	Dye concentration	Light source	Time of light exposure
$c-MoTe_2$	0.2 g/L	10 mg/L	500 W Xe lamp	90min
$a-MoTe_2$	0.2 g/L	10 mg/L	500 W Xe lamp	90 min
$a-MoTe_3$	0.2 g/L	10 mg/L	500 W Xe lamp	90 min
TiO_2/GO	4 g/L	0.01 mmol/L	450 W metal halide lamp	60 min
NCQDs/TiO_2	0.1 g/L	10 mg/L	300 W Xe lamp	60 min
TiO_2/Ag/rGO	0.2 g/L	20 mg/L	mercury lamp	75 min
TiO_2-MoS_2	0.2 g/L	50 mg/L	/	180 min
Zn/TiO_2	2 g/L	20 mg/L	visible light irradiation	60 min

Carbon Dots@TiO_{2-x}	0.4 g/L	20 mg/L	Xe lamp (<420 nm)	90min
